# Challenges of Decoding Transcription Factor Dynamics in Terms of Gene Regulation

**DOI:** 10.3390/cells7090132

**Published:** 2018-09-07

**Authors:** Erik W. Martin, Myong-Hee Sung

**Affiliations:** Transcription Systems Dynamics and Biology Unit, Laboratory of Molecular Biology and Immunology, National Institute on Aging, National Institutes of Health, Baltimore, MD 21224, USA; erik.martin@nih.gov

**Keywords:** transcription factors, gene expression, real-time dynamics, systems biology, microscopy, microfluidics, fluorescent reporters, smRNA-FISH, scRNA-seq

## Abstract

Technological advances are continually improving our ability to obtain more accurate views about the inner workings of biological systems. One such rapidly evolving area is single cell biology, and in particular gene expression and its regulation by transcription factors in response to intrinsic and extrinsic factors. Regarding the study of transcription factors, we discuss some of the promises and pitfalls associated with investigating how individual cells regulate gene expression through modulation of transcription factor activities. Specifically, we discuss four leading experimental approaches, the data that can be obtained from each, and important considerations that investigators should be aware of when drawing conclusions from such data.

## 1. Introduction

To perform essential biological functions and respond to changes in their microenvironments, cells systematically change the expression levels of process-relevant genes, while maintaining homeostatic expression of so-called housekeeping genes. For example, after detecting lipopolysaccharide (LPS), a bacteria-derived substance and molecular indicator of infection, macrophages alter the expression levels of several thousand genes, while maintaining baseline expression of other genes [1]. We still do not fully understand the mechanisms underlying how the cells of different tissues regulate the expression of such an astounding number of genes in a coordinated manner to respond to changes in their surroundings. The interaction of transcription factors (TFs) with DNA is fundamental for the regulation of gene expression [2]. Each of the more than 2600 human TF proteins [3] can influence transcription by interacting with specific DNA sequence motifs located throughout the chromatinscape. TFs are thought to not only determine which genes are expressed, but also influence the degree and timing of gene expression. However, their temporal activity patterns (hereafter referred to as TF dynamics) can be unexpectedly complex [4,5,6,7,8,9,10,11], which raises questions about their functional significance, and also complicates efforts to understand the role of TF dynamics in terms of gene regulation.

**Figure 1:**
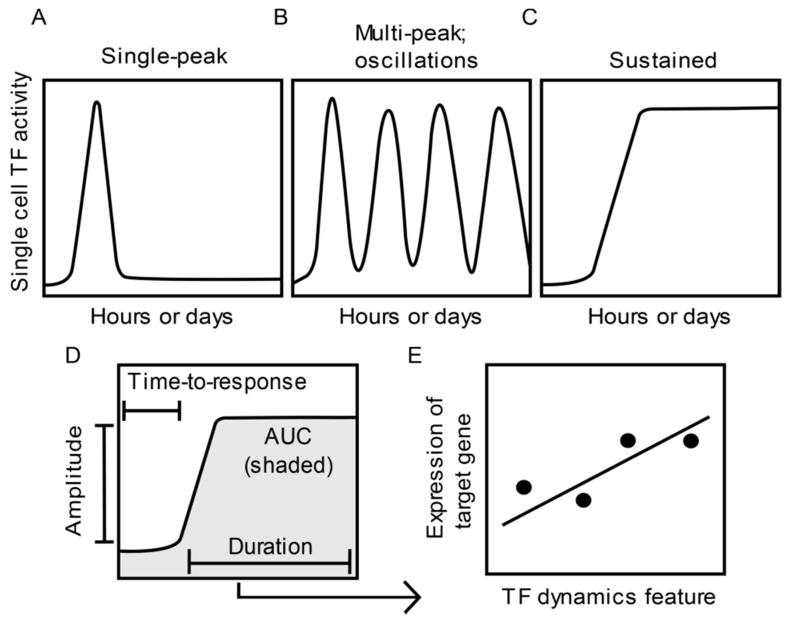
**Features of single cell transcription factor (TF) dynamics and their impact on gene expression.** (**A**–**C**) Example patterns of single cell TF dynamics observed in response to stimuli. (**D**) Quantitative features that characterize TF dynamics. (**E**) Assessment of the relationship between a feature of TF dynamics (**D**) and the expression of a target gene across different treatment or experimental groups.

In investigations of TF dynamics in single cells, TFs are often expressed as fluorescent fusion proteins and imaged via live-cell fluorescence microscopy. Imaging enables real-time recording of stimulus-induced changes in single cell TF abundance, nuclear localization, and residence time [12,13,14]. Patterns of signal-induced TF dynamics observed in mammalian cells include single peak, multiple peaks or oscillations, and sustained activation (Figure 1A–C). Through quantitative image analysis, specific features which describe various aspects of single cell TF dynamics can be extracted. For example, TF dynamics features, such as the length of time until TF activity is detected (time-to-response or onset delay), the amplitude or fold-change, the length of time a TF remains active (duration), and time-integrated TF activity (area under the curve (AUC)) [5] (Figure 1D), have been used to test for correlation with downstream readouts.

Changes in the population-level expression of specific target genes have been correlated to single cell dynamics of TFs, including nuclear factor κB (NF-κB) [13,15,16,17,18,19], SMAD [20], and p53 [21,22,23,24] (Figure 1E). However, TF dynamics-mediated regulation of mammalian gene expression at the single cell level has only been investigated in a limited number of seminal studies [23,25,26,27,28,29,30,31], partly due to technical constraints. These studies measured TF dynamics and gene expression in the same single cells by combining live cell imaging of a TF reporter with one of four experimental approaches: (1) single-molecule RNA fluorescence in situ hybridization [23,25,26]; (2) single-cell RNA-seq [27]; (3) imaging fluorescent reporter protein expression levels [13,29,30,31]; or (4) use of microfluidic immunoassays to measure levels of secreted proteins encoded by the induced genes [28]. 

## 2. smRNA-FISH

Single-molecule RNA fluorescence in situ hybridization (smRNA-FISH) is a molecular technique characterized by its ability to detect low levels (<5 copies per cell) of endogenous RNA transcripts of interest in individual cells [32,33]. It requires incubating fixed cells with fluorescently labeled probes which hybridize to complementary RNA transcripts. After hybridization, the abundance (and spatial distribution) of the fluorescent probe-RNA complexes are measured by fluorescence microscopy and serve as readouts of gene expression levels [32,33]. Although commonly performed to analyze the expression of a few genes, smRNA-FISH has been multiplexed to analyze the expression levels of tens to thousands of different genes [34,35,36,37,38]. Using this approach, certain dynamic features of NF-κB [25], p53 [23], and SMAD [26] have been revealed to correlate with the expression of their target genes in the same cells. Specifically, fold changes in the initial peak response of NF-κB [25] and SMAD [26] were found to correlate with target gene expression, while the duration of TF activity was important for p53-mediated gene expression [23]. 

**Figure 2:**
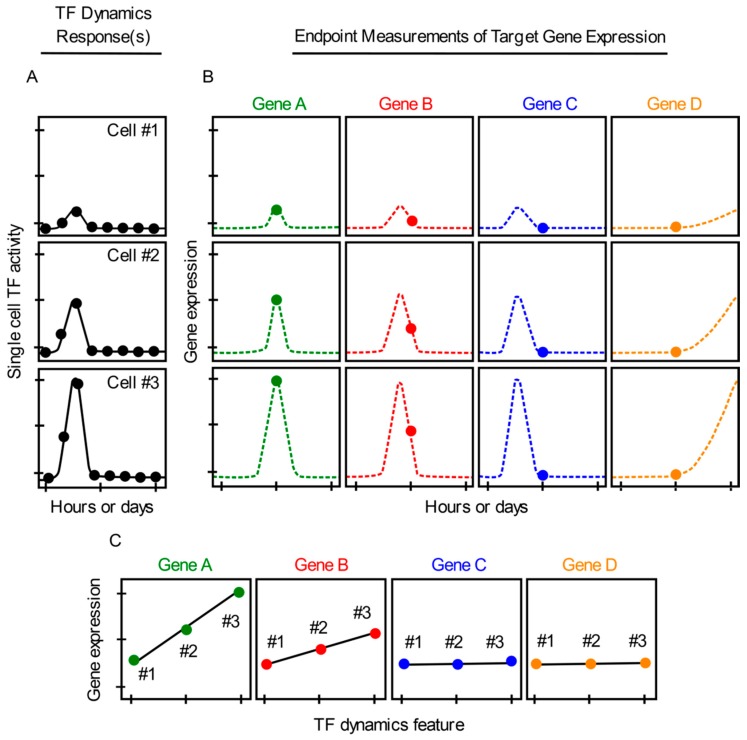
**Continuous measurements of TF dynamics and endpoint measurements of gene expression in the same single cells using smRNA-FISH or scRNA-seq.** (**A**) Single cell TF dynamic responses (cells #1–3) imaged continuously (dots) after a hypothetical cell activation. (**B**) Single-cell smRNA-FISH or scRNA-seq endpoint measurements (dots) of gene expression levels for hypothetical genes A–D induced by the TF dynamics in cells #1–3 (**A**). (**C**) Graphs depicting positive correlation (genes A, B) and spurious lack of correlation (genes C, D) (due to incomplete data) between TF dynamics and single cell gene expression. Dotted lines represent unsampled measurements.

Despite the high-resolution measurements of transcripts enabled by smRNA-FISH, this approach has a caveat which must be considered in the interpretation of results. smRNA-FISH cannot be used to monitor temporal changes in gene expression in the *same* single cells over time. Instead, only a single endpoint snapshot measurement of gene expression can be obtained per cell, after TF dynamics have been continuously imaged in each cell (Figure 2A,B). Therefore, it is difficult to adequately measure gene expression patterns or dynamics in single cells using smRNA-FISH. If only one snapshot of gene expression is taken per cell for a specific gene or set of genes, gene expression patterns that occur before or after the snapshot is taken are unable to be recorded. Subsequently, important data collection may be missed (Figure 2B), which could impact determinations as to whether TF dynamics correlate or do not correlate with single cell gene expression (Figure 2C). The use of one endpoint leaves this approach especially prone to false-negative conclusions (Figure 2C, genes C–D). If two or more timepoint snapshot measurements are recorded, conclusions may be strengthened incrementally. However, a negative result, i.e., a lack of correlation between TF dynamics and gene expression, would still be possible if the correlated behaviors occurred at some other (unsampled) times. 

## 3. scRNA-seq

Single-cell RNA sequencing (scRNA-seq) offers a uniquely powerful advantage which is orthogonal to smRNA-FISH, as this approach enables an unbiased profiling of gene expression. It measures the levels of endogenous RNA transcripts expressed in individual cells in a genome-wide manner by next-generation sequencing technology [39,40,41,42,43]. Such a wide coverage dataset cannot be obtained from other single cell approaches. To the best of our knowledge, scRNA-seq has only recently been used to study the impact of TF dynamics on single cell gene expression [27], where the investigators also employed smRNA-FISH to verify results for selected genes. This study revealed that LPS-induced patterns of single cell NF-κB dynamics (single narrow peak, sustained peak, or multiple peaks) correlate with the expression of distinct genes [27].

Unfortunately, scRNA-seq possesses the same pitfall as smRNA-FISH because it is also limited to providing only one single static snapshot of gene expression per cell (Figure 2B). scRNA-seq does not measure changes in gene expression in individual cells or capture transient or long-term gene expression events that take place before or after a snapshot measurement is obtained. Therefore, interpreting whether TF dynamics and single cell gene expression correlate with each other can also be problematic when using scRNA-seq (Figure 2A–C). Moreover, use of scRNA-seq currently requires that single cells be cultured and imaged for TF dynamics in isolation of each other, so that each cell can later be harvested for RNA analysis [27]. This blocks any naturally occurring exchange of paracrine signals between cells. Lastly, scRNA-seq comes with commonly acknowledged technical issues such as sparse data, noisy signals, amplification bias, and drop-outs [44].

## 4. Fluorescent Reporters

Measuring the expression of fluorescent reporter proteins in single cells is arguably the most information-rich approach for investigating the impact of TF dynamics on gene expression. Fluorescent reporter proteins, typically enhanced green fluorescent protein (EGFP) or alternatives, are expressed from customized gene expression cassettes introduced into cells by various means. The cassettes can be integrated into chromatin in cell lines and in vivo systems at random [45] or at specific locations [46], and their expression can be driven by a variety of TF-responsive promoters [47,48,49,50,51]. The abundance of the reporter protein is not an immediate readout of transcription, due to the time it takes for the translation of mRNAs, protein folding, and maturation of fluorescent proteins. The delay in the detection by microscopy can be minimized with careful reporter construct design and a fast-maturing fluorophore. After imaging data are collected, gene induction or repression can be quantified by the rate of change in the fluorescent intensity of the reporter [30,52,53].

**Figure 3:**
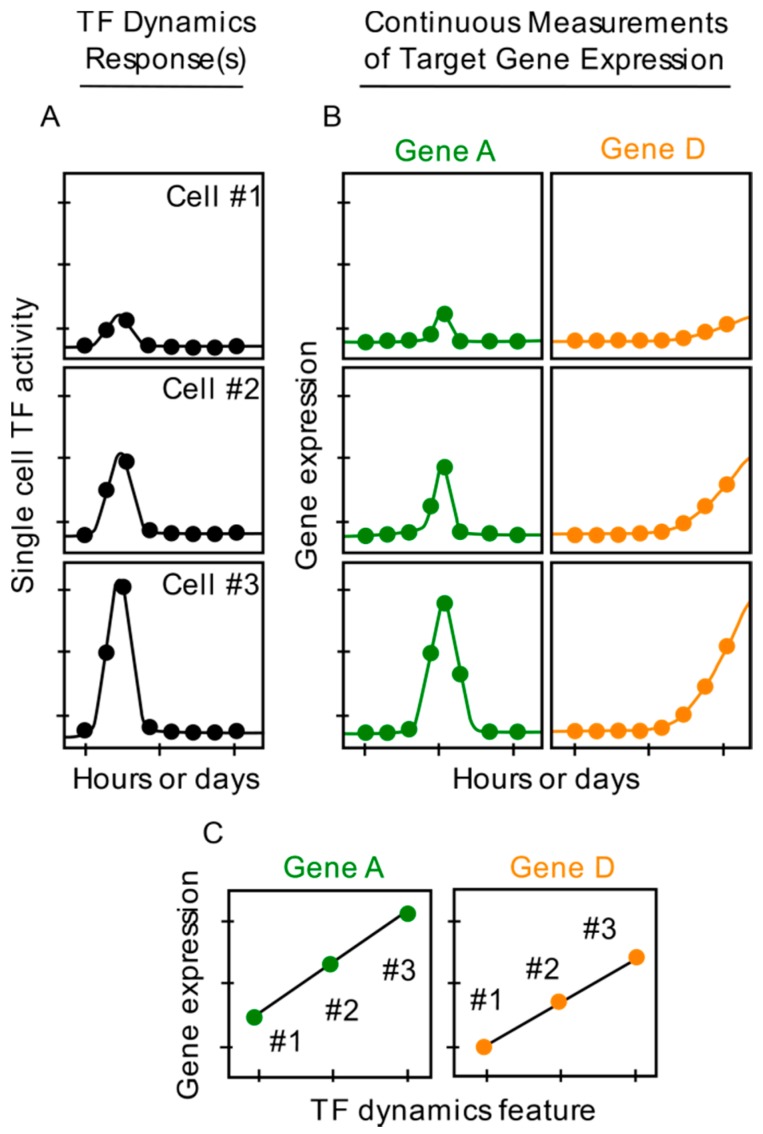
**Use of fluorescent reporter proteins enables continuous measurements of TF dynamics and gene expression in the same single cells.** (**A**). Single cell TF dynamic responses imaged continuously (dots) after hypothetical cell activation. (**B**). Continuous measurements (dots) of single cell gene expression levels for hypothetical genes A and D. (**C**). Graphs depicting correlation, due to sufficient data, between TF dynamics (**A**) and single cell expression levels of genes A and D (**B**). Positive correlation between TF dynamics and hypothetical genes B and C are not shown.

There are several advantages of using fluorescent reporter proteins to investigate single cell gene expression, especially as it relates to TF dynamics. First, expression levels of fluorescent reporter proteins can be measured in real-time at high temporal resolution, over many hours or even days (Figure 3B), without major disruptions to cell physiology or viability under optimal imaging conditions. A variety of unsynchronized expression patterns, including single or multiple bursts, can be captured, which are commonly missed by endpoint snapshot measurements (Figure 2B). Second, TF dynamics and gene expression can be imaged simultaneously in the *same* single cells by using fluorescent proteins with distinct excitation and emission wavelengths for the TF fusion protein and the reporter (Figure 3A,B). Capturing both gene expression dynamics and TF dynamics in the same single cells is critical to determining whether and which TF dynamic parameters impact gene expression at the single cell level. Third, the approach does not require culturing individual cells in isolated wells. Furthermore, through protein engineering, the stability and degradation rates of fluorescent reporter proteins can be modulated and characterized to calculate rates of reporter synthesis. Such calculations enhance the assessment of correlations between TF dynamics and single cell gene expression, and facilitate mathematical modeling of gene expression in single cells. An additional benefit of using fluorescent reporters is the ability to quantify absolute numbers of fluorescent TF proteins (and other important signaling molecules) through use of approaches including Fluorescent Correlation Spectroscopy [45,54,55].

Studies utilizing fluorescent reporter proteins to quantify gene expression and TF dynamics in the same single cells [13,29,30,31] have revealed the features of NF-κB dynamics which influence gene regulation. In macrophages, the nuclear intensity (abundance of NF-κB in the nucleus) was correlated with expression of a NF-κB-responsive tumor necrosis factor alpha (TNFα) promoter-driven mCherry reporter [30]. In HeLa cells, nuclear fold-change correlated with human immunodeficiency virus (HIV) long-terminal repeat (LTR) promoter-driven EGFP induction [29]. The number of peaks, i.e., the persistence of oscillations, correlated with the expression of IκBα-EGFP, driven by a 5X-κB-consensus-site promoter [13]. The time-to-response of interferon regulatory factor 7 (IRF7) has also been demonstrated to regulate the single cell expression of interferon beta (IFN-β) [31]. 

The use of fluorescent reporter proteins to measure single cell gene expression does have limitations. Unlike smRNA-FISH and scRNA-seq, the fluorescent proteins do not report expression of endogenous RNA transcripts. To date, we are unaware of studies that have investigated TF dynamics-mediated regulation of endogenous gene loci using this approach. In addition, due to constraints associated with the overlap of fluorescent protein excitation and emission spectra, only a few reporter proteins can be simultaneously imaged as readouts for expression of different genes. Detection of signals from fluorescent reporters is likely to be not as sensitive as smRNA-FISH for low-abundance transcripts. Imaging very low-abundance fluorescent reporters may require using an increased laser power, which can result in cellular toxicity if not addressed by adjusting other image acquisition parameters.

## 5. Microfluidic Immunoassays

A marked alternative to assessing gene expression through measurements of RNA transcripts or intracellular protein abundance is measuring the amount of induced proteins that are secreted from single cells. Different research groups have engineered microfluidic devices capable of culturing single cells in isolation, stimulating them individually, and then capturing and quantifying the levels of specific proteins that are secreted by each single cell via antibody-based immunoassays [56,57,58,59], even up to once every minute for extended time-courses [60]. When coupled with imaging systems, microfluidic immunoassays enable real-time measurements of single cell TF dynamics and protein secretion as a readout of single cell gene expression [56,59]. A major benefit of microfluidics is the throughput of data acquisition; the devices often contain tens or hundreds of individual cell culture and treatment channels, enabling multiplex treatments of individual cells with different ligands, ligand concentrations, and stimulus patterns. This multiplexed stimulation and detection capability vastly expands the capacity to study TF dynamics and single cell gene expression responses in a variety of different conditions. Moreover, microfluidics (combined with immunoassays or other single cell gene expression methods) enable studies of TF dynamics and gene expression in non-adherent cells, a feat not easily achieved using other approaches. A recent study combined imaging TF dynamics with microfluidic immunoassay of single cell protein secretion to study the impact of TF dynamics on single cell gene expression, and reported that NF-κB dynamics did not correlate with the expression of TNF-α protein [28]. This finding was contrary to what was observed for expression of a fluorescent reporter protein driven by the TNFα promoter [30], suggesting that the secretion of cytokines may be governed by additional regulatory steps regarding their release into the extracellular space. 

The fact that separate biochemical processes underlie the regulation of gene expression and protein secretion is probably the most significant pitfall associated with utilizing secreted proteins as measures of gene expression. It is not difficult to envision a case where single cell gene expression correlates with the dynamics of a TF, but the kinetics and extent of single cell protein secretion do not. Another caveat of the approach, which is shared by scRNA-seq, is the requirement for strict isolation of single cells in individual culture wells or channels. When interpreting data or comparing results with those of other studies, these points should be taken into consideration. 

## 6. Conclusions

Advances in microscopy, high-throughput sequencing, and microfluidics are steadily making the study of single cell TF dynamics more tractable. However, many unresolved and technically challenging issues remain regarding the fundamental aspects of TF dynamics and their role in gene regulation. Each of the approaches to measuring single cell gene expression discussed here, when coupled with imaging TF dynamics, has its own benefits and pitfalls. Any of the given methods may be the most appropriate depending on specific research needs. Nonetheless, we argue that the use of fluorescent reporter proteins is the technique which may well be the best option currently, being the only approach that provides real-time data in the same single cells. Such information-rich data can be used to determine per-cell correlations between TF dynamics and gene expression, which provides critical information for mechanistic modeling of the relevant regulatory processes. We look forward to improvements in existing technologies and the development of new methods that will facilitate our abilities to simultaneously measure real-time TF dynamics as well as expression of many endogenous genes in the same cells, in vitro and in vivo.
